# On-Chip Optical Trapping with High NA Metasurfaces

**DOI:** 10.1021/acsphotonics.2c01986

**Published:** 2023-03-15

**Authors:** Jianling Xiao, Tomasz Plaskocinski, Mohammad Biabanifard, Saydulla Persheyev, Andrea Di Falco

**Affiliations:** School of Physics and Astronomy, University of St. Andrews, North Haugh, St. Andrews, Fife, KY16 9SS, United Kingdom

**Keywords:** optical tweezers, metasurface, high NA, metalens, holography, lab-on-chip

## Abstract

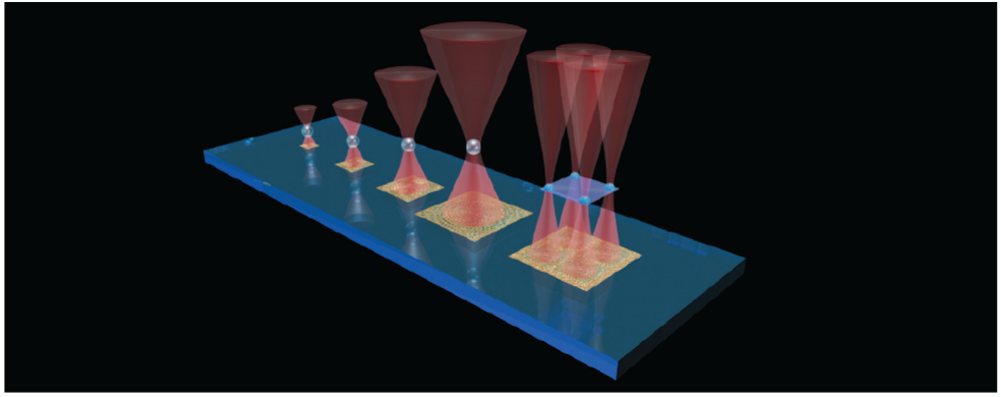

Optical trapping of small particles typically requires the use of high NA microscope objectives. Photonic metasurfaces are an attractive alternative to create strongly focused beams for optical trapping applications in an integrated platform. Here, we report on the design, fabrication, and characterization of optical metasurfaces with a numerical aperture up to 1.2 and trapping stiffness greater than 400 pN/μm/W. We demonstrate that these metasurfaces perform as well as microscope objectives with the same numerical aperture. We systematically analyze the impact of the metasurface dimension on the trapping performance and show efficient trapping with metasurfaces with an area as small as 0.001 mm^2^. Finally, we demonstrate the versatility of the platform by designing metasurfaces able to create multisite optical tweezers for the trapping of extended objects.

## Introduction

Optical trapping is a well-established experimental method for the noninvasive manipulation of microscopic objects. Generally, trapping of spherical particles with dimensions comparable to the wavelength of the used light occurs at the waist of a strongly focused beam. Here, a potential minimum exists where the scattering forces balance the restoring gradient forces.^1−3^ The technique can be extended to the simultaneous manipulation of multiple objects, creating a suitable optical landscape by engineering the phase and the amplitude of the light, e.g., via spatial light modulators (SLMs),^4^ digital micromirror devices,^5,6^ or interferometry.^7^ Using these methods, it is also possible to manipulate microscopic objects of complex shapes to perform advanced functions,^8−10^ including for applications in biology and medicine.^11,12^ Optical manipulation is now routinely used for particle sorting,^13,14^ rotating objects,^15,16^ nanowire assembly,^17,18^ cell stretching,^19,20^ and interrogation of biological specimens at the single molecule level.^21,22^

These techniques all rely on the use of bulky and expensive objectives with high numerical aperture (NA), to focus light strongly enough to create the required optical field gradient, to obtain strong and stable trapping. One of the key figures of merit in optical tweezing is the trap stiffness. This parameter represents the value of the spring constant of the damped oscillator, which describes the trapping potential.^23^ For higher trapping stiffness, trapped objects experience smaller displacements over time. Modern lab-on-chip solutions for optical trapping and manipulation propose to replace the use of these objectives with integrated lenses based on metasurface (MS) technology. Metalenses consist of 2D ensembles of subwavelength dielectric or metallic nanofeatures (meta-atoms) that can implement a desired optical function with extreme versatility. They can be used to control the parameters of the scattered light, including its phase,^24−27^ amplitude,^28−30^ polarization,^31−33^ and wavelength.^34,35^ Using a tailored meta-atoms distribution, it is possible to produce metalenses with NA operating at the theoretical diffraction limit.^36−39^

In the context of optical tweezing, the versatility in the design of MSs has been exploited to create polarization switchable lenses able to drag and drop particles in a two-dimensional plane. This silicon-based MS had a moderate NA = 0.6 with a stiffness *K* = 7.53 pN/μm/W for polystyrene particles with a diameter of 4.5 μm.^40^ A similar platform was used to create metalenses with NA = 0.56 to trap polystyrene beads with a diameter of 2 μm and a trapping stiffness around 13.54 and 33.70 pN/μm/W along the *x* and *y* directions, optimizing the shape of the optical beam at its focus point.^41^ A plasmonic-based bifocal MS was designed to trap particles with a diameter of 2 μm at two different distances from the lens plane, with NA = 0.7 (0.56) at a distance of 3 μm (7 μm), and with a trapping stiffness along the *x* and *y* axis of 7 (9) and 8 (13) pN/μm/W, respectively.^42^ Adapted Fresnel metalenses with NA = 0.88 were also patterned on the tip of optical fibers for trapping applications, with a stiffness of 100 pN/μm/W.^43^ Metamaterials technology has been employed beyond the use of metalenses to create suitable optical potentials for optical trapping and manipulations, comprehensibly reviewed in ref (44).

**Figure 1:**
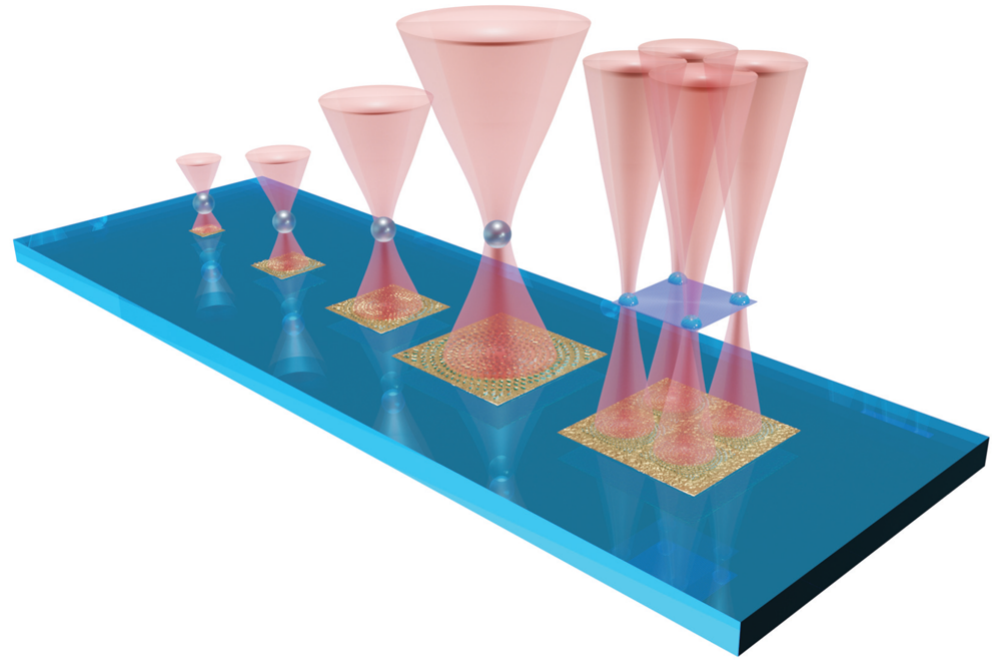
Concept of reflective-type MSs for light-trapping applications.

In this paper, we present the design, fabrication, and characterization of reflective type MSs with a NA of 1.2 and a trap stiffness up to 430 pN/μm/W, comparable with the stiffness obtained with a bulky microscope objective with the same NA. Additionally, for a given NA, we tested MSs with areas from 900 μm^2^ to ∼0.09 mm^2^ and a trapping distance varying from 7.1 to 75 μm. To design the MSs we choose metal–insulator–metal three-layers Pancharatnam-Berry (PB) meta-atoms, with circularly polarized light at a wavelength λ = 830 nm. We also demonstrate that the MSs can be designed to create multiple foci to trap nonspherical objects, such as fishnet membranes, decorated with trapping handles (see Figure 1).

## Design of Hologram and Meta-Atoms

**Figure 2:**
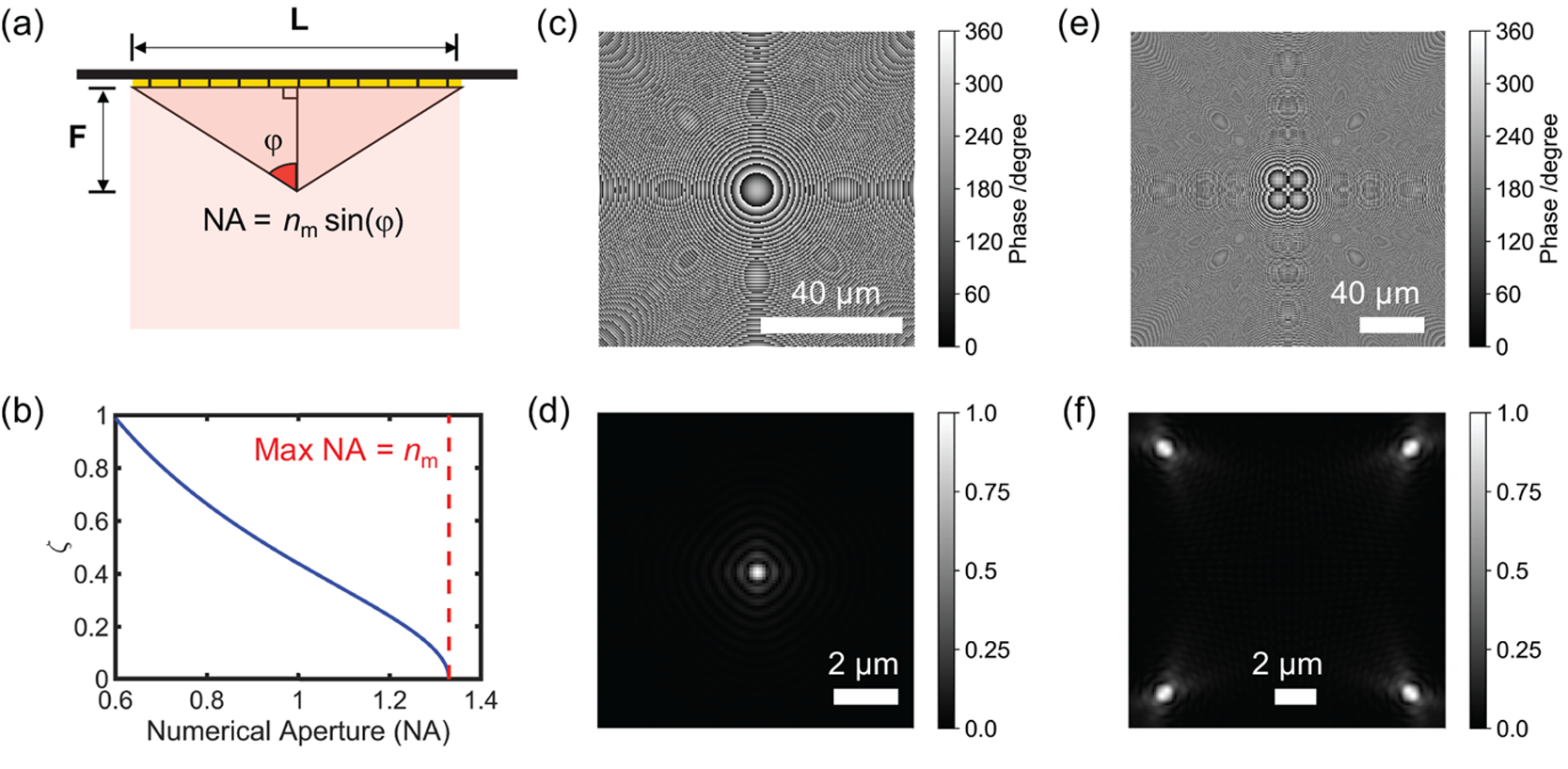
(a) Schematic illustration of the trapping geometry. (b) Dependence of ζ from NA. The red dashed line indicates the maximum achievable NA, which is equal to the refractive index of the medium (here, *n*_m_ = 1.33). (c) Simulated phase profile and corresponding holographic image (d), for MS with a side length 90 μm. (e) Phase profile and (f) holographic image for multifoci MS, with a side length of 200 μm.

Given the requirement to create MSs with multiple trapping sites, the MSs were designed using the Gerchberg–Saxton algorithm,^45^ rather than using a simple analytical phase profile. For a given focal length, the maximum area of the metalens and the periodicity of the meta-atoms impose boundaries on the NA of the lens, following the Nyquist sampling theorem.^46^ As detailed below, here we used a periodicity *P* that does not limit the NA for the wavelength used and the refractive index of the medium. From a practical point of view, it is instructive to obtain the maximum trapping distance *F* in terms of a lens side length *L*, which would guarantee the desired NA = *n*_m_ sin(φ), where *n*_m_ is the refractive index of the medium and φ is the half angle of the focused beam, as shown in Figure 2a. For this purpose, it is convenient to define the geometrical parameter . Figure 2b shows how ζ is related to the NA. In the following, we specifically focused on six different MS geometries, summarized in Table 1.

**Table 1:** Summary of MS Parameters

NA	*L* (μm)	*F* (μm)	ζ	no. of pixels in hologram	trapping points
1.2	30	7.1	0.2	100 × 100	1
1.2	60	14.3	0.2	200 × 200	1
1.2	90	21	0.2	300 × 300	1
1.2	210	50	0.2	700 × 700	1
1.2	315	75	0.2	1050 × 1050	1
1.3	300	32	0.1	1000 × 1000	1
∼1.2	200	47	0.2	667 × 667	4

Figure 2c,d shows the simulated phase profiles of the hologram and the corresponding holographic image for a MS with an area of 90 μm × 90 μm, with NA = 1.2, producing a single trapping spot at distance of around 21 μm from the MS. Figure 2e is the simulated phase profile for a MS with area 200 μm × 200 μm, creating four trapping points at a distance of 47 μm from the MS, each with NA approximately equal to 1.2, which is used to trap extended objects.

**Figure 3:**
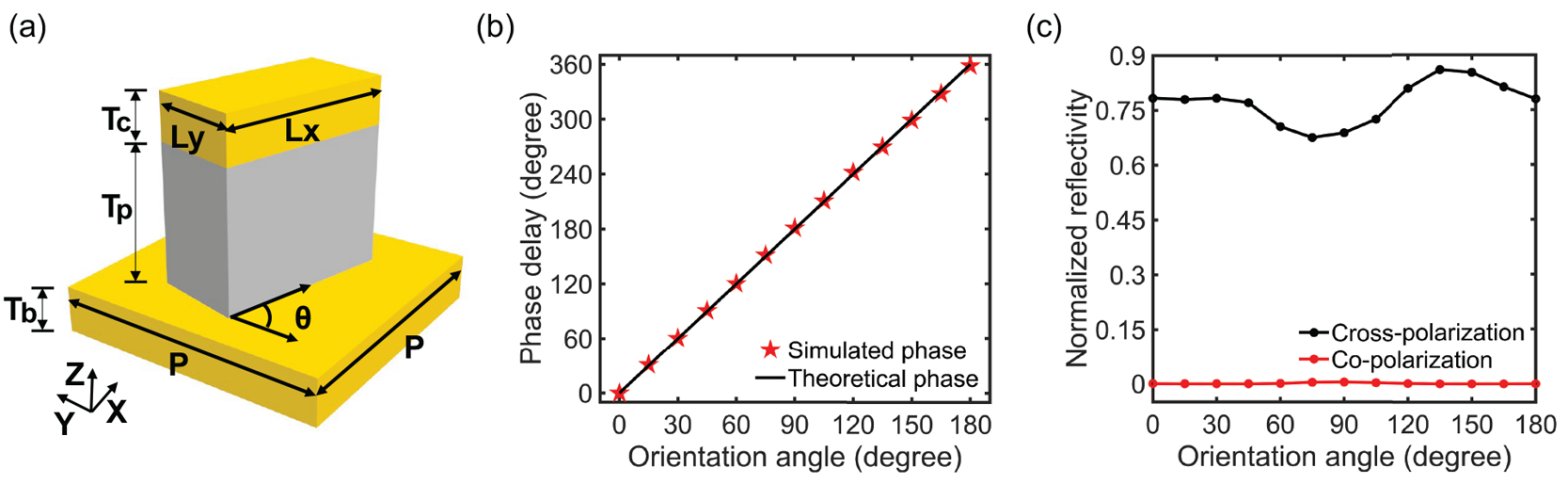
(a) Schematic image of the unit cell. (b) Comparison of simulated phase delay (red star) and theoretical PB phase (black line) when the orientation angle θ varies from 0 to 180°. (c) Simulated cross-polarization (black) and copolarization (red) reflectivity with normal incidence when the orientation angle θ varies from 0 to 180°.

The holograms were discretized in 12 phase levels and implemented physically using nanorods with different orientation angles θ. The schematic image of a unit nanorod is shown in Figure 3a, where the dielectric pillars are sandwiched by a top cap and bottom gold layer.^47^

The response of the meta-atoms was modeled using CST Studio Suite, with unit cell boundary condition applied along the *x*/*y* axes. Along the *z*-axis, for the port we used open boundary conditions and perfect electric conducting boundary conditions for the bottom port. The surrounding medium was modeled as water. The MS was designed to work for circular polarization, with reflected light cross polarized with respect to the incident beam. The geometrical parameters of the unit cell, shown in Figure 3a, were optimized to maximize the reflectivity, taking into account the limited resolution of the fabrication. The parameter *P* = 300 nm is the periodicity, *T*_p_ = 160 nm is the pillar thickness, *L*_*x*_ = 220 nm, and *L*_*y*_ = 90 nm are the optimized length and width of the top cap, respectively (see also Figure S1 in the Supporting Information), *T*_c_ = 40 nm is the thickness of the top cap, and *T*_b_ = 190 nm is the thickness of the bottom gold layer.

Figure 3b shows the comparison of the numerically simulated phase delay and of the theoretical phase, when the orientation angle θ varies from 0 to 180°. Figure 3c shows the simulated cross-polarization and copolarization reflectivity at normal light incidence against θ.

**Figure 4:**
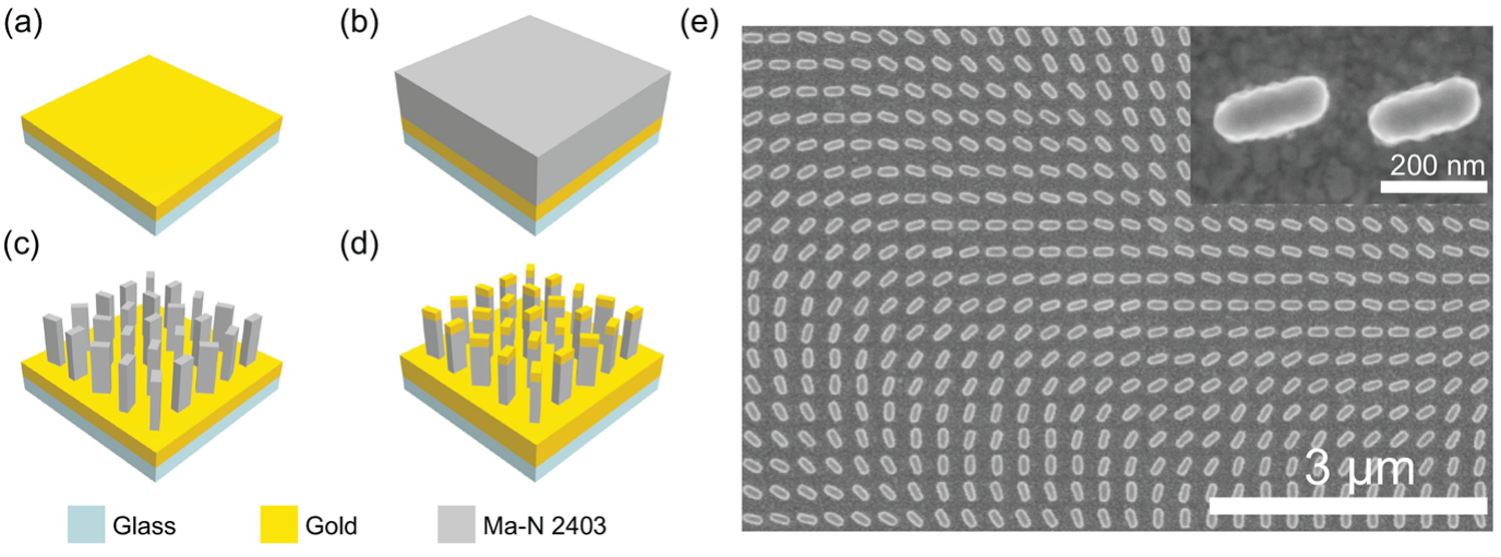
(a–d) Schematic image of the fabrication process. (a) Deposition of NiCr and gold via an e-beam evaporation; (b) Spin-coating of Ma-N 2403 film; (c) Pattern definition via electron beam lithography (EBL) and development; (d) Deposition of a gold cap. (e) SEM image of fabricated MS. The inset shows a zoomed-in image of the nanorods.

Figure 4a–d shows the schematic images of the fabrication process and Figure 4e shows the scanning electron microscope (SEM) image of a typical MS. More details about the fabrication can be found in the Experimental Section.

## Results and Discussion

**Figure 5:**
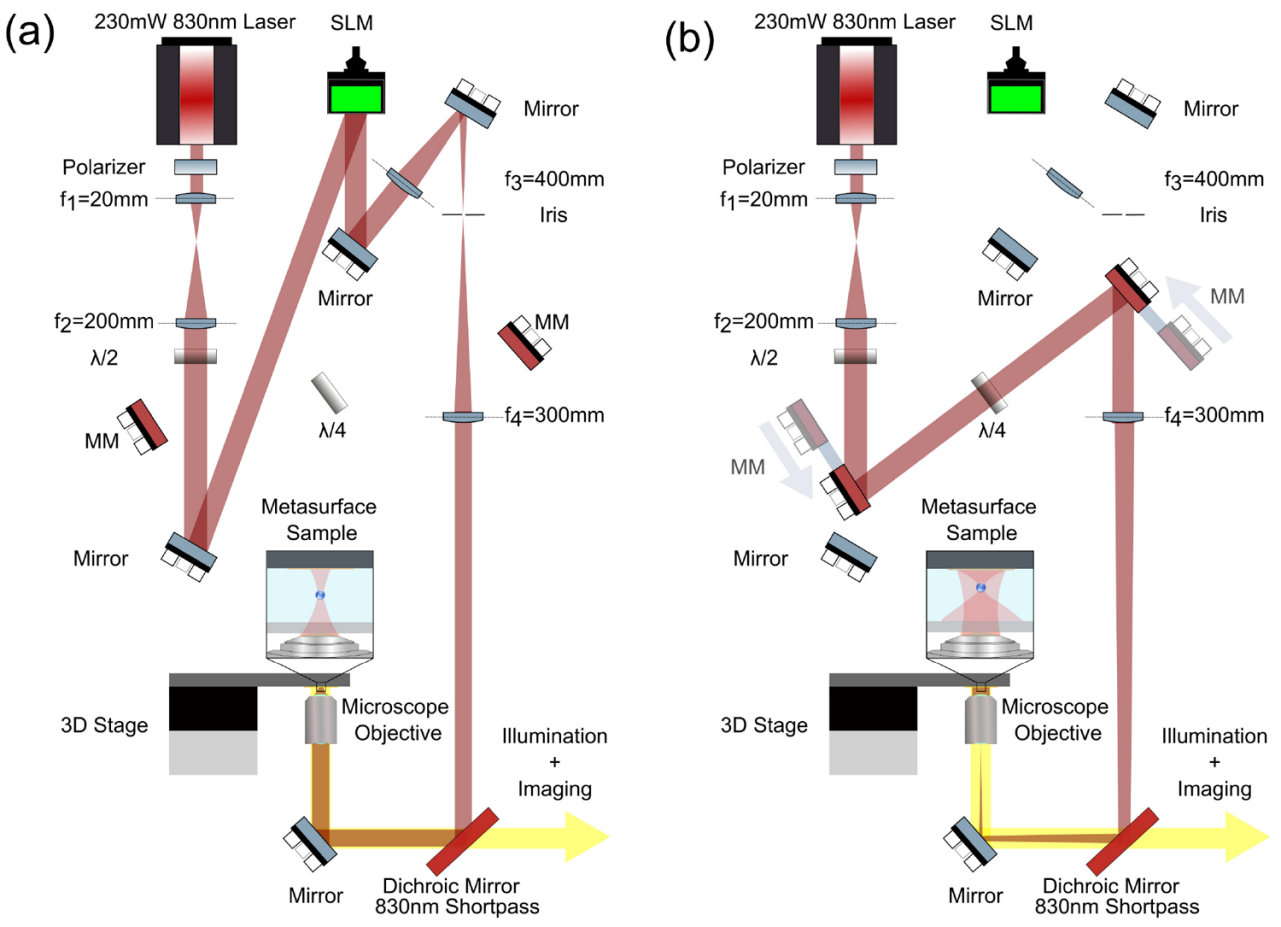
Schematic image of the optical setup. (a) SLM-enabled holographic tweezing system. (b) MSs-based holographic tweezing system.

The diagram of the setup is shown in Figure 5. For the trapping source, we used a CW Omicron LuxX laser of the wavelength λ = 830 nm with a maximum output power of 230 mW. The laser beam was expanded with a 10× telescope made by the lenses f_1_ and f_2_, and its polarization was aligned to that required by the SLM with a polarizer, followed by a λ/2 waveplate. Two computer-controlled movable mirrors (MM) delivered light to two distinct pathways. The first route included an SLM (Meadowlark 1920 × 1152), followed by a 4f system, made by the plano-convex lenses f_3_ and f_4_, a dichroic mirror and a water immersion Olympus 60× (NA = 1.2) microscope objective. The back focal plane of the objective was therefore conjugated with the SLM plane and able to create a holographic trap landscape on the sample plane. The other route focused the beam via f_4_ on the back focal plane of the objective, thus delivering a collimated beam to the sample of size ∼200 μm, to illuminate the whole MS. In this case, we included a λ/4 waveplate to convert the beam to a circular polarization, as required for the correct use of the MSs. Switching between the two pathways facilitated the transfer of the objects from the SLM-enabled tweezers to the trapping sites of the MSs. The switching took less than 1 s. The sample was prepared by suspending the microparticles in heavy water (D_2_O) in a chamber made by a 100 μm thick vinyl spacer, sandwiched between the glass slide with the MSs and a 170 μm thick coverslip. The sample was mounted on a Piezo 3D stage to control the position along the *x*/*y*/*z* direction. The paths for the illumination (a collimated white LED) and image forming (a lens with 150 mm focal length, followed by a USB Basler CCD camera aceA640-750) were both placed on the other side of the dichroic mirror (not shown).

For all the quantitative measurements we used latex beads with diameter *d* = 2 μm. To determine the performance of the optical traps we used the same bead, first trapped with the SLM/objective tweezers and then transferred systematically to all the measured MSs. This improved the consistency and quality of the characterization. The position trajectories of the particle were extracted from 10 s long videos acquired at 1000 fps, for different laser powers (measured by placing a power meter above the front lens of the microscope objective), using a well-established approach, based on the shift properties of the Fourier transform.^48^

Each measurement was repeated four times for the MSs and two times for the SLM traps. The trap stiffness in the plane perpendicular to the beam propagation (*k*_⊥_) was obtained averaging the values of the stiffness along the *x* and *y* directions. As customary, *k*_*x*,*y*_ = 12π^2^η*rf*_*x*,*y*_, with *f*_*x*,*y*_ being the corner frequency of the Lorentzian fitting the power density spectrum of the trajectory dynamics in *x* and *y*, η = 1.2 mPa is the viscosity of heavy water at room temperature, and *r* is the radius of the particle. For the sake of simplicity, in all cases, we considered that the particle was trapped in the middle of the chamber.

**Figure 6:**
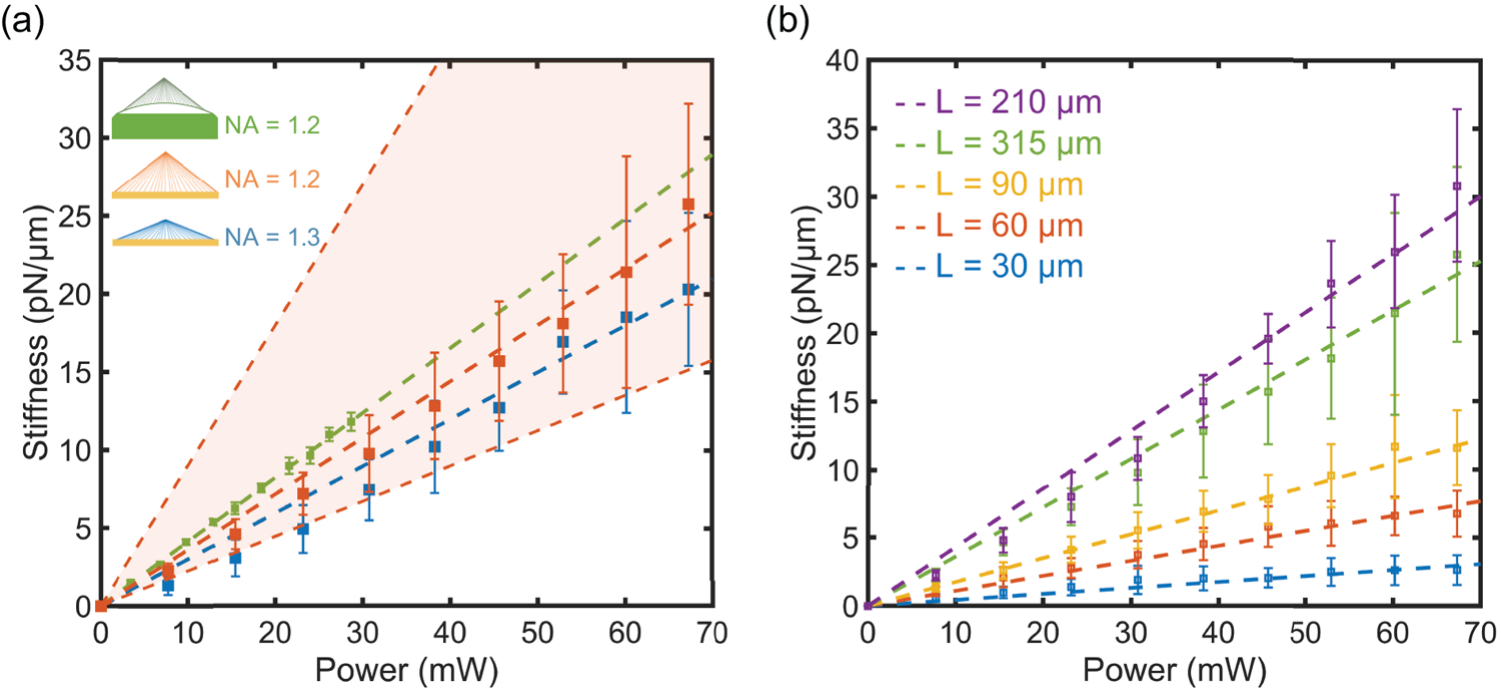
(a) Trapping stiffness vs input power for the microscope objective with NA = 1.2 (green), the MS with NA = 1.2 (orange), and the MS with NA = 1.3 (blue). For the MS we assumed a 50% efficiency. The shaded area indicates the trap stiffness for efficiency going from 20% to 80% for the NA = 1.2 case. (b) Comparison of trapping stiffness of MSs with NA = 1.2 with different side lengths.

Figure 6a shows a comparison of the trap stiffness for MSs designed to have NA = 1.2 and 1.3, both with a side length of ∼300 μm, with that of the trap obtained using the SLM and the objective. To this end, the values of the trapping power were scaled assuming the MSs have a diffraction efficiency of 50%. While it would be practically challenging to measure quantitatively the efficiency, due to the light collection requirement, we note that this value is rather conservative, for common experimental realizations of reflective type metasurfaces.^47,49^ To quantify the effect of the efficiency on the evaluation of the trap stiffness, we added a shaded region in Figure 6a, for the MS with NA = 1.2, with an efficiency between 20% (higher boundary) and 80% (lower boundary). Further discussions on the efficiency are included in the Supporting Information. The dashed lines are the linear fit of the measurements.

**Figure 7:**
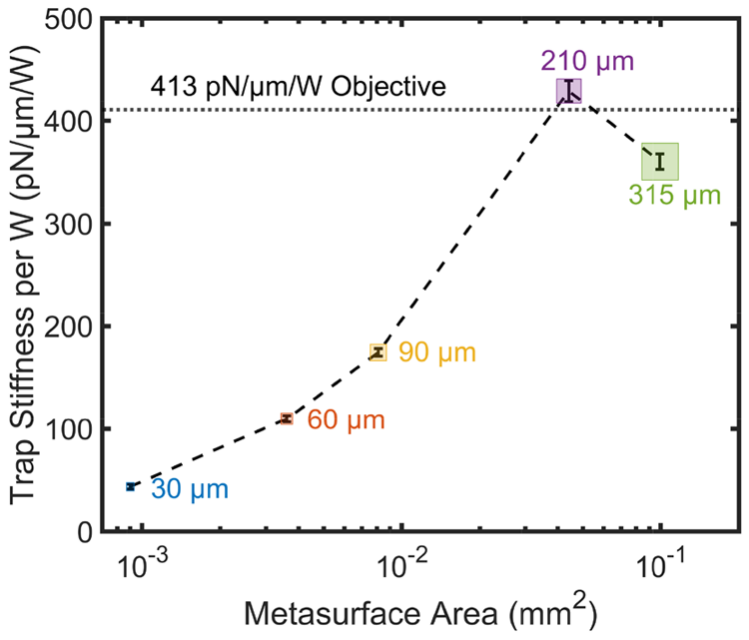
Comparison of trapping stiffness per W of MSs with different sizes. The dot-dashed line shows the same figure of merit for the microscope objective.

The results shown in Figure 6 demonstrate that the MSs produce a trap stiffness comparable to that of an objective with the same NA. It is not surprising that the MS with NA = 1.3 exhibits a lower stiffness than the MS with lower NA and the objective, since the diffraction efficiency of the meta-atoms reduces at very large angles, unless optimized designs are adopted^38^ (see also the directivity of the meta-atoms in Figure S2 in the Supporting Information). This clearly means that for the case NA = 1.3, the effective value of the NA is smaller than that of the designed lens. It should also be noted that in both these cases, the collimated beam does not fill the whole MS. To explore systematically the dependence of the trapping quality from the geometrical parameters of the MSs, we used the design with NA = 1.2 of Table 1. Figure 6b shows that the trap stiffness appears to increase with the area of the MSs, until a maximum value, before decreasing. In order to prevent scaling artifacts due to the fact that the smaller MSs are much smaller than the illuminating beam, we chose to present the results not normalized to the area of the MSs. This produces the impression that smaller MSs are less efficient, which is not necessarily the case. This choice rather underestimates the efficiency of the MSs. This is further supported by Figure S3 in the Supporting Information, which shows the comparison of the numerical and experimental beam spot profiles formed by the MSs, showing that the size of the smaller MSs does not degrade the quality of the focused spot. Additionally, it can be noticed that since the MS with side length *L* = 210 μm has a size comparable to the illuminating beam diameter, its trap stiffness exhibits a slight nonlinearity at lower powers. The viability of the MS-enabled platform as a replacement of bulky, high NA objectives is better appreciated in Figure 7, where we plot the trap stiffness per unit incident power (given by the gradients of the linear fits) for all the cases, along with the efficiency of the trap obtained with the objective (dot-dashed line). Remarkably, this means that our platform performs well even with MSs with area smaller than 0.001 mm^2^. Figure 7 also shows that the largest MS produces a lower trap stiffness, which is due to a combination of under-filling and reduced diffraction efficiency of the meta-atoms at the edges of the MS, as anticipated when discussing the results of Figure 6a.

Additionally, it is interesting to note that the trapping efficiency of the MSs remained sufficiently high even for severely misaligned illumination (shown in the Supporting Information, Movie S1). Figure S4 in the Supporting Information shows the measured trapping stiffness when the particles were trapped at the corners of the field of view of the camera.

It is important to notice that although our MSs work in reflection using meta-atoms with metallic features, the optical trapping potential is not determined by the local hot spots at the corners of the nanopillars. In our case, the particles are trapped up to tens of microns away from the surface of the MS. This geometrical configuration is substantially different from arrangements where thermal effects play a role, both in the near field of plasmonic resonators^50^ and in the proximity of a metallic film.^51,52^ This is further confirmed by the results of Figure S4, where we show that the particles can be trapped in offset locations with respect to the center of the incident beam. We can therefore exclude meaningful thermophoretic effects in the trapping dynamics presented here.

**Figure 8:**
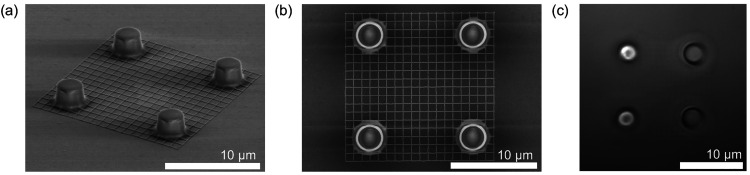
(a) and (b) SEM images of a SU8 fishnet of area 15 × 15 μm^2^. (c) Still frame from the video of the fishnet membrane trapped by a multisite MS.

These high quality MSs are ideally suited for on-chip trapping platforms. This is also the reason why we chose to design them with square form factors, to improve the packing efficiency. An alternative route is to consider the unique capabilities of holographic MSs to encode complex optical functions, e.g., to trap extended objects. Figure 8a,b shows the SEM images of a polymeric fishnet membrane fabricated via e-beam lithography and decorated with integrated handles for optical manipulation. This class of extended objects is extremely promising for biophotonic applications due to their intrinsic optomechanical stability.^10^Figure 8c shows the same membrane trapped by the multisite MS shown in Figure 2e,f. Supporting Information, Movie S2 shows the handover of the trapped membrane from the SLM/objective traps to the MS traps. The detailed fabrication process of the fishnet membrane can be found in the Experimental Section.

These proof of principle experiments demonstrate the viability of a new biophotonic platform, e.g., for the optical equivalent of a surgical theater, where biological objects of interest and microscopic tools, such as force probes,^53^ rotators^54^ and waveguides^55^ can be trapped and manipulated as required.

## Conclusion

In this paper, we presented the design, fabrication, and characterization of MSs for optical trapping and manipulation, with efficiency comparable with that of high NA objectives, with a trap stiffness per unit power up to 430 pN/μm/W. The results were based on reflective-type holographic MSs with high NA, with a minimum area smaller than 0.001 mm^2^. The versatility of the platform was further demonstrated creating a tailored holographic MS to provide a multisite trapping potential, which we used to hold in place a large fishnet membrane. This demonstrates that holographic MSs enable a new class of multifunction devices for lab-on-chip applications.

## Experimental Section

### Sample Fabrication

MSs: A 1 mm thick glass slide was cleaned in 10 min ultrasonic baths both in acetone and isopropanol, dried, and treated with O_2_ plasma for 3 min. A 150 nm thick layer of gold with 3 nm of NiCr adhesion layer was deposited using an electron beam evaporator (Edward AUTO306). A 160 nm thick layer of Ma-N 2403 (Micro Resist Technology) was spin-coated onto the gold layer at 4000 rpm for 30 s and prebaked for 15 min at 90 °C. The nanorod patterns were defined by a Raith e-Line Plus EBL system with dose 250 μC/cm^2^. After the exposure, the sample was postbaked at 90 °C for 10 min, developed in Ma-D (Micro Resist Technology) for 95 s, and washed in deionized water for 30 s. Finally, a 40 nm thick layer of gold was deposited on the sample to form the top cap.

Fishnet membrane: The silicon substrate was cleaned in 10 min ultrasonic baths both in acetone and isopropanol, dried, and treated with O_2_ plasma for 3 min. A 100 nm thick layer of Omnicoat (Microchem) was spin-coated on the wafer at 1000 rpm for 45 s as the sacrificial layer and baked at 230 °C for 60 s. A 90 nm thick SU8 (Microchem) was then spin-coated at 5000 rpm for 60 s, followed by a soft-baking step at 65 °C for 1 min and 95 °C for 4 min. The fishnet patterns and alignment markers were first defined by EBL exposure with 5 μC/cm^2^. After the exposure, the sample was postbaked at 100 °C for 2 min and developed in ethyl lactate for 45 s. After the development, the alignment markers on the sample were covered by dicing tape before spin-coating a 2 μm thick SU8, at 1000 rpm for 60 s. After removing the tape, the sample was baked at 65 and 95 °C each for 5 min. The second EBL exposure defined the handles with a dose of 5 μC/cm^2^. After exposure, the sample was post-baked and developed with the same parameters as before. A tetramethylammonium hydroxide-based solvent was used to remove the Omnicoat to release the fishnet membrane from the silicon substrate.

Sample preparation for trapping: The microfluidic chip was prepared by placing an adhesive vinyl spacer with a thickness of 100 μm with a 1 cm hole in the middle to create a well around the metasurfaces. The hole was filled with a solution of D_2_O and beads, a glass coverslip was placed on top, and the resulting chamber was sealed using fast-drying nail polish.

## Data Availability

The data underlying this study are openly available in the Data Set of the University of St. Andrews Research Portal at 10.17630/e1745c6c-3042-4b14-be26-5506fe2ca247.

## Supporting Information Available

Method for the measurement of the MS efficiency, reflectivity and directivity of the nanorods, evaluation of the trapping spot profile, trapping stiffness for different illumination conditions, and bead size (PDF)
Movie S1: Video of the trapped particle (MP4)
Movie S2: Video of the handover of the trapped fishnet membrane (MP4)
