# Supplementary material for: On-Chip Optical Trapping with High NA Metasurfaces
Source: ACS Photonics. 2023 Mar 15;10(5):1341–8. doi: 10.1021/acsphotonics.2c01986 (PMC10197168; doi:10.1021/acsphotonics.2c01986)
Supplement: Supplementary file 1 — ph2c01986_si_001.pdf [file ph2c01986_si_001.pdf]

Supporting Information
On chip optical trapping with high NA metasurfaces

Jianling Xiao,[†,‡] Tomasz Plaskocinski,[†,‡] Mohammad Biabanifard,[†] Saydulla Persheyev,[†] and Andrea Di Falco[*,†]

†*School of Physics and Astronomy, University of St Andrews, North Haugh, St. Andrews, Fife, KY16 9SS, United Kingdom*

‡*These authors contributed equally to this work.*

E-mail: adf10@st-andrews.ac.uk

Phone: +44(0)1334 463108. Fax: +44(0)1334 463104

The file includes:

- Methods: Measurement of the MS efficiency.
- Supporting Figures: S1 - S5

  Figure S1: Reflectivity of the nano-rods vs Lx and Ly in water.

  Figure S2: Directivity vs angle at normal incidence along the long and short axis of the nanorod.

  Figure S3: Comparison of the numerical and experimental beam spot profiles formed by the MSs with different side length.

  Figure S4: Calculation of the trap stiffness at different positions.

  Figure S5: Comparison of trapping stiffness of 2 µm and 0.5 µm silica beads.

- Movie: Movie S1 and Movie S2

  Movie S1: Video of a 5 µm latex particle trapped by a metasurface.

  Movie S2: Video of the handover of the trapped fishnet membrane from the SLM/objetive tweezers to the metasurface-enabled tweezers.

Methods: measurement of the MS efficiency

An indicative measure of the MSs diffraction efficiency can be obtained generating a collimated beam instead of a focused spot. To this end, we fabricated a sample with a side length of 315 µm to form a 1 mm diameter dot, 10 cm away from the metasurface at an angle of 12 degrees. The sample was submerged in water during the experiment. The wavelength of the incident circularly polarized beam was 830 nm with a diameter of 267 µm, to ensure the underfilling of the metasurface. The incident power and reflected power were 23 mW and 3.1 mW, which were collected by power meter resulting in a calculated efficiency of 13.4%.

Supporting Figures

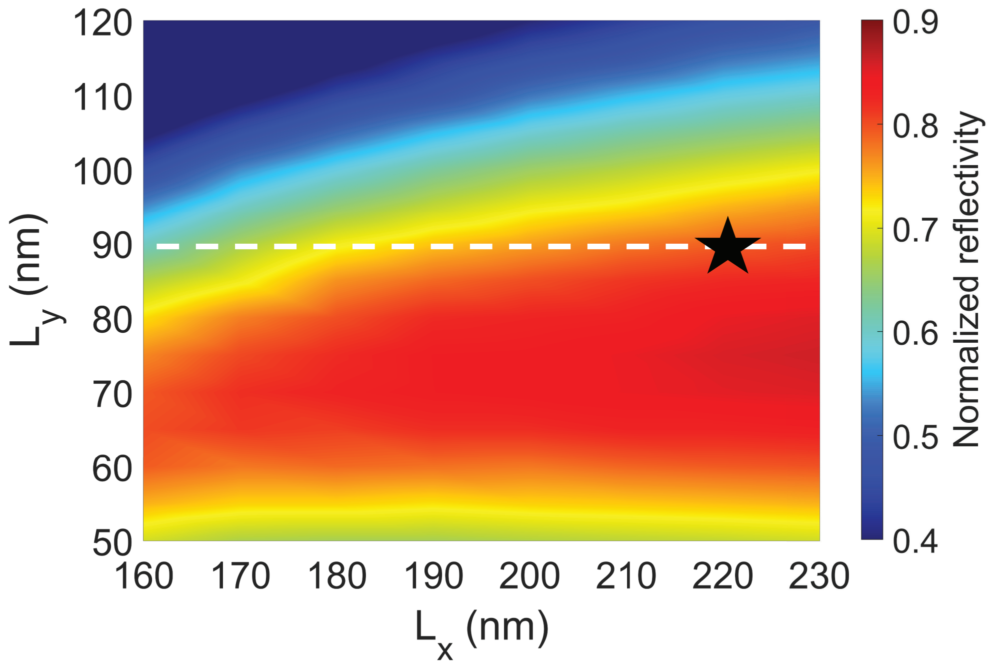

Figure S1: Reflectivity of the nano-rods vs L_x and L_y in water. In these simulations, P = 300 nm, T_p = 160 nm and T_c = 40 nm. The black star indicates the chosen design parameters for the fabrication. The dashed white line denotes the fabrication limit for the definition of high quality nanorods.

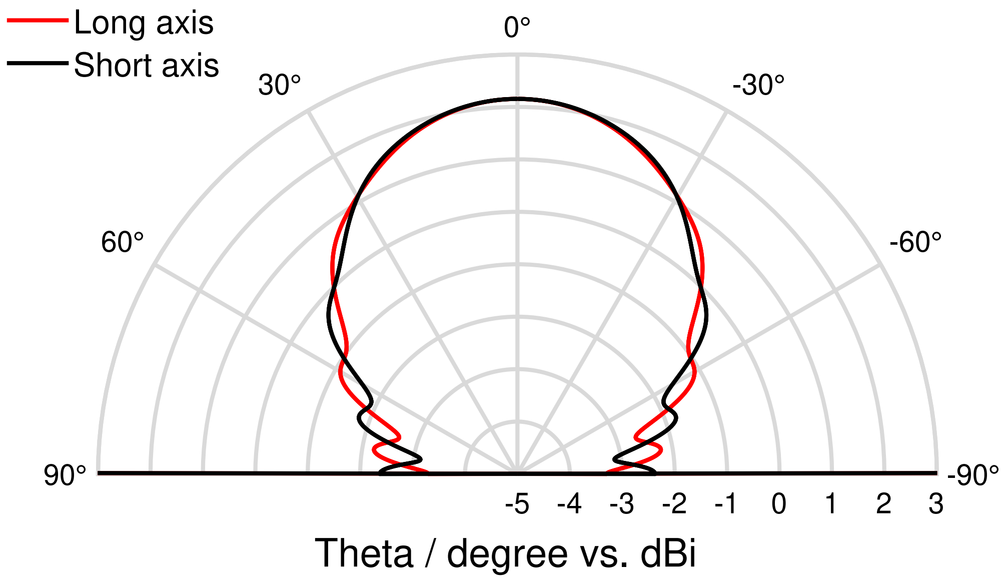

Figure S2: Directivity vs angle at normal incidence along the long and short axis of the nanorod. The main lobe magnitude is 2.15 dBi and the average angular width (3 dB) along the long and short is 107 deg.

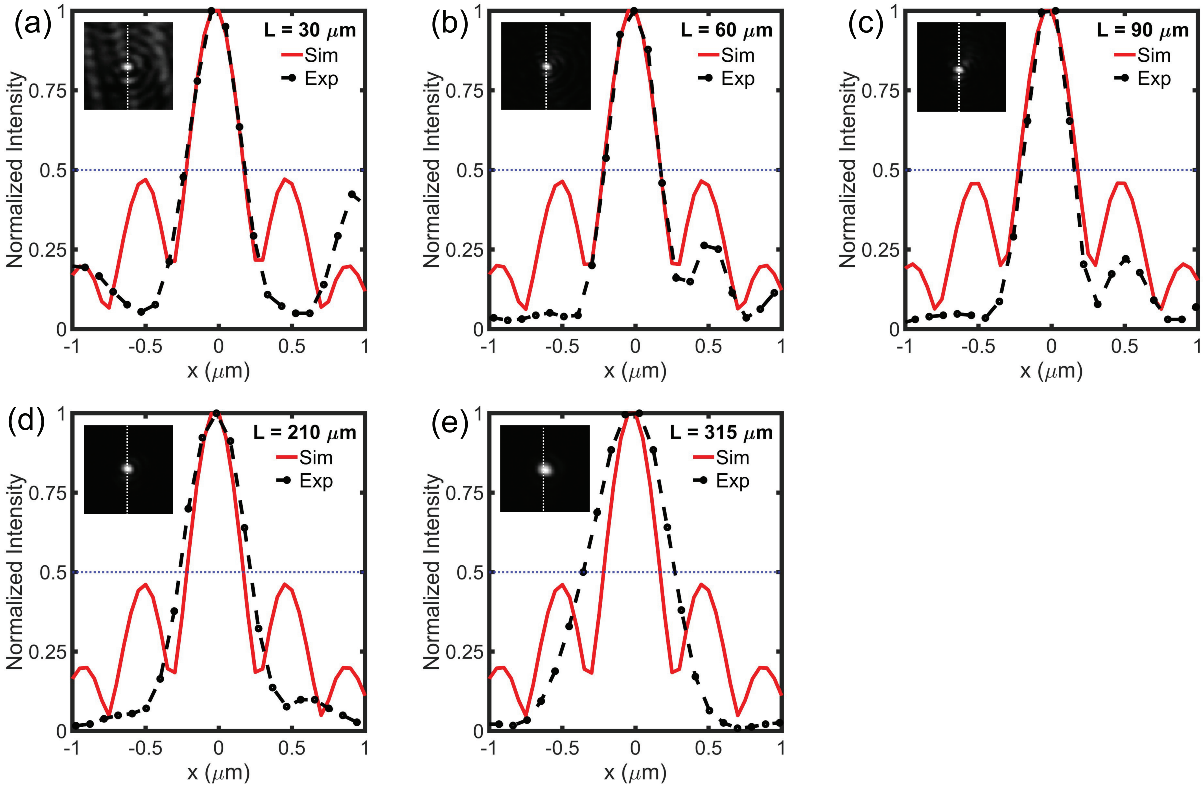

Figure S3: Comparison of the numerical and experimental beam spot profiles formed by the MSs with different side length. (a) 30 µm, (b) 60 µm, (c) 90 µm, (d) 210 µm and (e) 315 µm. The side lobes are a numerical artefact, due to the limited resolution of the numerical method used to generate the holographic spots.

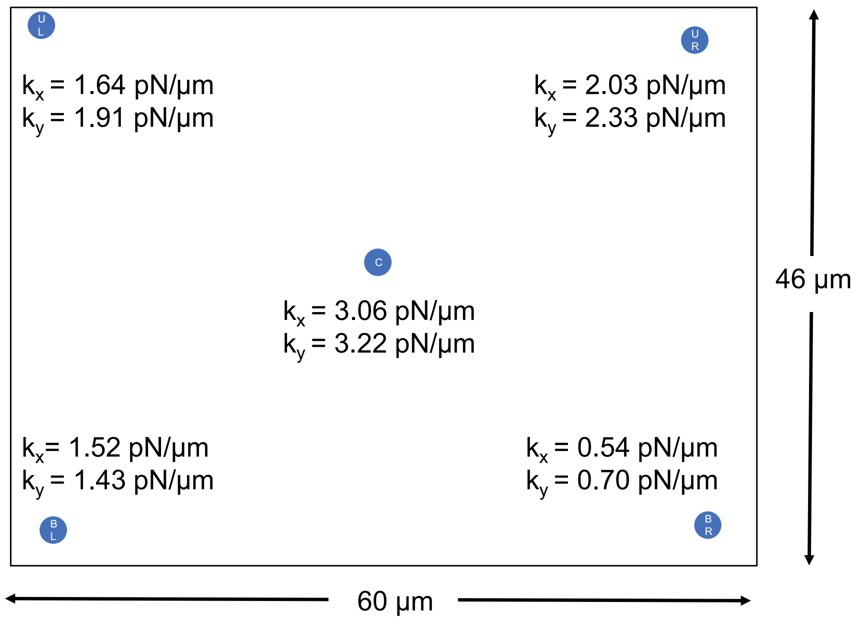

Figure S4: Calculation of the trap stiffness at different positions, relative to the centre of the camera.

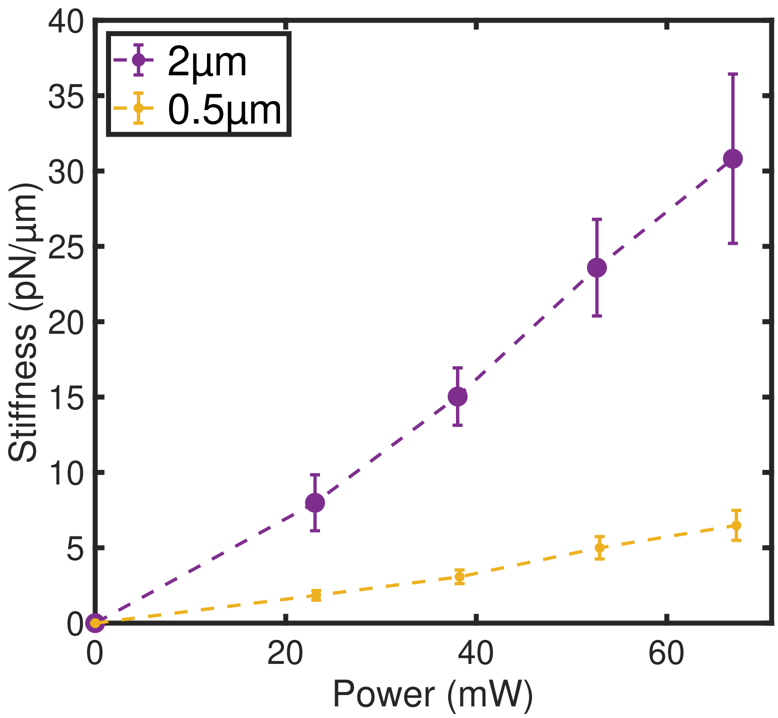

Figure S5: Comparison of trapping stiffness of 2 µm and 0.5 µm silica beads using a MS with side length L = 210 µm, F = 50 µm and design NA of 1.2.
